# Study on the cross-resistance of *Aedes albopictus* (Skuse) (Diptera: Culicidae) to deltamethrin and pyriproxyfen

**DOI:** 10.1186/s13071-024-06485-1

**Published:** 2024-09-27

**Authors:** Ling-qun Lin, Ya-hui Chen, Yi-fan Tian, Yu-sen Chen, Zhao-yang Zheng, Jing-xin Wu, Fen Hu, Cheng Wu, Li-Hua Xie

**Affiliations:** Department of Pathogenic Biology, School of Basic Medical Sciences, Fujian Medical University, No. 1 Xuefu North Road, Fuzhou, 350122 Fujian China

**Keywords:** *Aedes albopictus*, Cross-resistance, Genic mutation, Deltamethrin, Pyriproxyfen

## Abstract

**Background:**

Insecticide resistance poses a significant challenge in the implementation of vector-borne disease control strategies. We have assessed the resistance levels of *Aedes albopictus* to deltamethrin and pyriproxyfen (PPF) in Fujian Province (China) and investigated the correlation between these resistance levels and mutations in the voltage-gated sodium channel (VGSC).

**Methods:**

The WHO bioassay protocol was used to evaluate the resistance coefficient of *Ae. albopictus* to deltamethrin and PPF, comparing a susceptible population from the Foshan (FS) area with wild populations from the Sanming (SM), Quanzhou (QZ), Zhangzhou (ZZ), Putian (PT) and Fuzhou (FZ) areas in Fujian Province. Genomic DNA was analyzed by PCR and sequencing to detect knockdown resistance (*kdr*) in the VGSC, specifically at the pyrethroid resistance alleles V1016V, I1532I and F1534F. Molecular docking was also performed to analyze the binding interactions of PPF and its metabolite 4'-OH-PPF to cytochrome P450 (CYP) 2C19, 2C9 and 3A4 and *Ae. albopictus* methoprene-tolerant receptors (AeMet), respectively.

**Results:**

The analysis of resistance to deltamethrin and PPF among *Ae. albopictus* populations from the various regions revealed that except for the sensitive population in FS and the SM population, the remaining four regional populations demonstrated resistance levels ranging from 4.31- to 18.87-fold for deltamethrin and from 2.85– to 3.62-fold for PPF. Specifically, the FZ and PT populations exhibited high resistance to deltamethrin, whereas the ZZ and QZ populations approached moderate resistance levels. Also, the resistance of the FZ, PT and ZZ populations to PPF increased slowly but consistently with the increasing trend of deltamethrin resistance. Genomic analysis identified multiple non-synonymous mutations within the VGSC gene; the F1534S and F1534L mutations showed significant resistance to deltamethrin in *Ae. albopictus*. Molecular docking results revealed that PPF and its metabolite 4'-OH-PPF bind to the *Ae. albopictus* AeMet receptor and CYP2C19.

**Conclusions:**

The wild *Ae. albopictus* populations of Fujian Province showed varying degrees of resistance to deltamethrin and PPF and a trend of cross-resistance to deltamethrin and PPF. Increased vigilance is needed for potential higher levels of cross-resistance, especially in the PT and FZ regions.

**Graphical Abstract:**

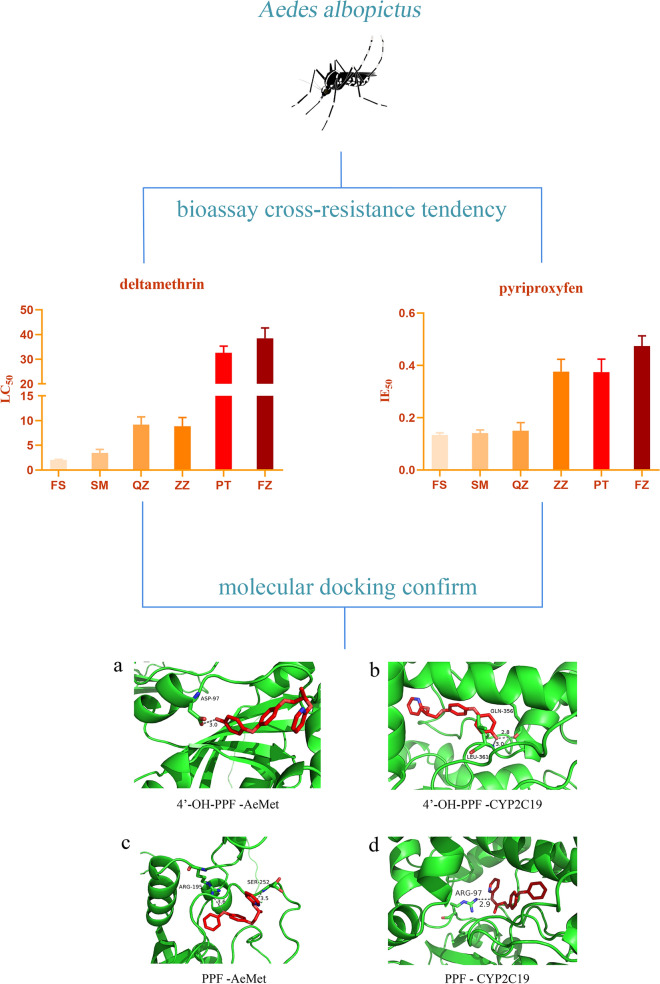

**Supplementary Information:**

The online version contains supplementary material available at 10.1186/s13071-024-06485-1.

## Background

*Aedes albopictus*, commonly referred to as the Asian tiger mosquito, is a significant vector species that is widely distributed across southern China. This mosquito species plays a pivotal role in the transmission of diseases such as dengue fever, Zika virus and chikungunya fever [1–4], most notably in Fujian Province, which is one of the high-incidence areas of dengue fever in China [5, 6]. In recent years, dengue fever outbreaks have occurred frequently, posing serious public health challenges [7]. Chemical control is currently the primary means to manage *Ae. albopictus* populations and mitigate the risk of dengue fever [8–10]. However, the frequent and indiscriminate application of insecticides has resulted in the development of resistance in *Ae. albopictus*, compromising control efforts [11].

In the current landscape of vector control, the arsenal of insecticides predominantly comprise pyrethroids, carbamates, organophosphates, organochlorines and insect growth regulators. Among these, pyrethroids, carbamates and organophosphates are frequently utilized in the management of *Ae. albopictus* populations [12]. However, statistical data have revealed varying degrees of pyrethroid resistance in *Ae. albopictus* across numerous regions in China, attributed to the widespread usage of deltamethrin, with resistance in the larval stage observed to be more pronounced than that in the adult stage [13]. The underlying mechanism of resistance to deltamethrin in *Ae. albopictus* is intricately associated with mutations in the voltage-gated sodium channel (VGSC), encoded by the VGSC gene, which is the primary target of pyrethroid insecticides. Mutations in the VGSC gene can reduce its affinity for insecticides, leading to resistance [14, 15]. This resistance is prominently characterized by the lack of knockdown effect after insecticide exposure, and is therefore also known as knockdown resistance (*kdr*) [16]. In comparison, metabolic resistance refers to the enhanced activity of detoxification enzymes or the increase in the copy number of genes involved in metabolism, resulting in accelerated insecticide detoxification. Detoxification enzyme systems primarily include cytochromes P450 (P450s or CYPs). Increased expression of P450 genes and amino acid mutations are crucial mechanisms through which insects develop resistance [17, 18]. Specifically, mutations such as V1016V, I1532I and F1534F on the VGSC gene have been identified as significant contributors to pyrethroid resistance, as evidenced by recent research findings [19–22].

Recently, juvenile hormone (JH) has garnered significant attention as an environmentally benign insecticide, owing to its selective toxicity towards arthropods [23, 24]. Pyriproxyfen (PPF), an analog of JH, exhibits high activity against a variety of disease-transmitting insects [25]. It is therefore worth exploring the resistance levels of *Ae. albopictus* both to deltamethrin and PPF in the wild populations of Fujian Province, as well as to ascertain the existence of any cross-resistance.

At the molecular level, molecular docking is widely recognized as one of the most popular and effective structure-based computational methods used to predict interactions between molecules and biological targets [26]. Typically, this process begins by predicting the spatial orientation of a ligand within a receptor and subsequently to assessing their complementarity using a scoring function [26]. Molecular docking technology can also be used to explore the interaction resistance between deltamethrin and PPF at the molecular level.

To evaluate the resistance levels of *Ae. albopictus* to deltamethrin and PPF in Fujian Province and analyze the relationship between these resistance levels and mutations in the VGSC, we collected *Ae. albopictus* samples from various locations in Fujian Province, including Sanming (SM), Quanzhou (QZ), Zhangzhou (ZZ), Putian (PT), and Fuzhou (FZ). Our primary objectives were twofold: (i) to determine the extent of resistance levels to deltamethrin and PPF; and (ii) to analyze the correlation between resistance and mutations in the VGSC, with a specific focus on the *kdr* gene. This study provides valuable data on mutations on the *kdr* gene in the wild populations of *Ae. albopictus* in China, thereby establishing a foundation for effective resistance detection and the development of targeted control strategies.

## Methods

### Mosquito collection and rearing

Larval samples of *Ae. albopictus* were collected using mosquito ovitraps placed in various habitats, such as shade, flowerpots or green belt areas, across a number of different cities in Fujian Province, from May to October 2022. Sampling information is provided in Additional file 1: Data file S1. The mosquito ovitraps were collected and transported to the laboratory for morphological identification. The larvae were reared to adulthood and provided blood meals to facilitate egg production for the subsequent generation's development. Specifically, third- and fourth-stage larvae (L3, L4, respectively) of the F1-F2 generation were used in the tests with pyrethroids and PPF. The sensitive *Ae. albopictus* Foshan (FS) population (generously provided by Professor Chen Xiaoguang's research group at Southern Medical University) was used as the control. The breeding room was maintained at 26 ± 2 °C and a relative humidity at 70 ± 10% under a 16/8-h light/dark photoregimen

### Bioassay experiments

The pre-experiment involved setting up seven to nine different concentrations of pesticides to initially determine the range of insecticide concentration corresponding to mortality rates of between 10% and 90%. The formal experiment tested five concentration gradients within this range. The experiment was replicated 3 times. The experimental group comprised 25 L3/L4 *Ae. albopictus* larvae placed in a solution of 99 ml of dechlorinated tap water and 1 ml of different concentrations of insecticide solution. The control group consisted of 25 L3/L4 *Ae. albopictus* larvae placed in a solution of 99 ml of dechlorinated tap water and 1 ml of acetone. For deltamethrin, the number of dead larvae was counted 24 h after the experiment, and the half-maximal lethal concentration (LC_50_; concentration of deltamethrin required to cause mortality in 50% of the larvae) was calculated. For PPF, emergence inhibition was measured daily until all of the larvae or pupae had died or emerged into adults, and the half-maximal inhibition of emergence (IE_50_; the concentration of pyriproxyfen needed to inhibit 50% of larval emergence) was calculated.

### Determination of resistance to deltamethrin and PPF in *Ae. albopictus* larvae

We used larvae as recommended by the WHO for assessing larval resistance (https://www.who.int/publications/i/item/WHO-ZIKV-VC-16.1). Probit analysis was used to calculate the two critical parameters: the LC_50_ and the IE_50_.

### Death determination and correction for interference factors

To assess whether the larvae or pupae were dead, we touched the larva or pupa gently with a suction pipe. If there was no active escape behavior from this stimulation, they were assessed as dead.

Interference factors needed to be corrected for. For the deltamethrin experiments, if > 10% of the control larvae pupated during the course of the experiment, the test was discarded and repeated. If the mortality in the control group was between 5% and 20%, the mortalities of the treated groups were corrected according to Abbott’s formula. For PPF, if adult emergence in the control was < 90%, the test was discarded and repeated; when adult emergence was between 91% and 99%, the data were corrected using Abbott’s formula, as shown:

$${\text{Corrected mortality }}\left( {\text{\% }} \right)\;{ = }\;\frac{{{\text{\% mortality in test }} - {\text{\% mortality in control}}}}{{{100 } - {\text{\% mortality in control}}}}\, \times {100}$$

Resistance ratios (RR) are often calculated and are useful indices by which to monitor the evolution of insecticide resistance in a field population. This ratio helps quantify the level of resistance in the field population compared to the susceptible population. The calculation of RR involves dividing the LC_50_ (or IE_50_) of a field population by that of a susceptible population, as shown:

- RR (deltamethrin) = LC_50_ (field population)/LC_50_ (susceptible population)
- RR (PPF) = IE_50_ (field population)/IE_50_ (susceptible population).

When RR is < 5, the field population is considered to be susceptible; when RR is between 5 and 10, mosquitoes are considered to have moderate resistance; and when RR is > 10, the mosquitoes are highly resistant (https://www.who.int/publications/i/item/WHO-ZIKV-VC-16.1).

### Genomic DNA extraction and mutation analysis of three representative alleles of the VGSC gene

Genomic DNA was extracted from a single adult *Ae. albopictus* specimen using a DNA extraction kit (Adlai Biotechnology, Beijing, China). The extracted DNA served as a template for amplifying fragments corresponding to domains II and III of VGSC. Primers were synthesized according to published protocols [20]. The PCR reaction mixture comprised PCR Master Mix (12.5 μl), 10 μmol/l forward and reverse primers (2 μl each), template DNA (5.5 μl) and double distilled water, up to a volume of 25 μl. The PCR cycling conditions consisted of an initial denaturation at 94 °C for 3 min; 35 cycles of denaturation at 94 °C for 30 s, annealing at 55 °C (for domain II) or 53 °C (for domain III) for 30 s and extension at 72 °C for 30 s; with a final extension step at 72 °C for 10 min. PCR products with clear bands and no tail were subjected to gene sequencing by Shangya Company after electrophoresis in a 1% agarose gel. Sequence alignment and mutation analysis were performed using MEGA (v. 11.0.13) and Chromas (v. 2.6.5).

### Molecular docking of PPF and metabolites with CYPs and met receptors of *Ae. albopictus*, respectively

To explore the binding interactions of PPF and its metabolite 4’-OH-PPF with specific receptors in *Ae. albopictus*, we conducted molecular docking studies. The chemical structure of 4′-OH PPF was obtained from PubChem (https://pubchem.ncbi.nlm.nih.gov/). We focused on CYP2C19, CYP2C9 and CYP3A4 in the CYP450 family for the molecular docking study [27]. Given the absence of resolved protein structures for mosquito CYP enzymes, we selected homologous CYP enzymes from* Homo sapiens* that are known for their involvement in drug metabolism, and aligned our choice with available data from PubChem. Next, we searched the Protein Data Bank (PDB) for three-dimensional crystal structures from *Homo sapiens* CYP2C19, CYP2C9 and CYP3A4 receptors (PDB 4GQS, 1R9O and 3NXU, respectively) (https://www.rcsb.org/pdb/home/sitemap.do). The predicted amino acid sequences of the* Met* gene of *Ae. albopictus* were found in Vectorbase (https://vectorbase.org/vectorbase/app), and the structure of *Met* was predicted by AlphaFold (https://alphafold.ebi.ac.uk/). Specifically, we used the crystal structure-labeled CYP2C19 from the PDB for virtual docking. Molecular docking simulations were performed with AutoDock Vina software. The solvent molecules and original ligands in the crystal structures were removed in the docking calculations. All structural figures were generated with the PyMOL molecular visualization system.

### Statistical analysis

Probit-regression analysis of the mortality and IE data obtained at each concentration was performed to determine the LC_50_ and IE_50_ valued using SPSS version 25.0 (IBM Corp., Armonk, NY, USA) according to published protocols [28]. The results were plotted using GraphPad Prism version 9.0 (GraphPad Software, San Diego, CA, USA).

## Results

### Resistance of *Ae. albopictus* to deltamethrin in some cities of Fujian Province

**Fig. 1:**
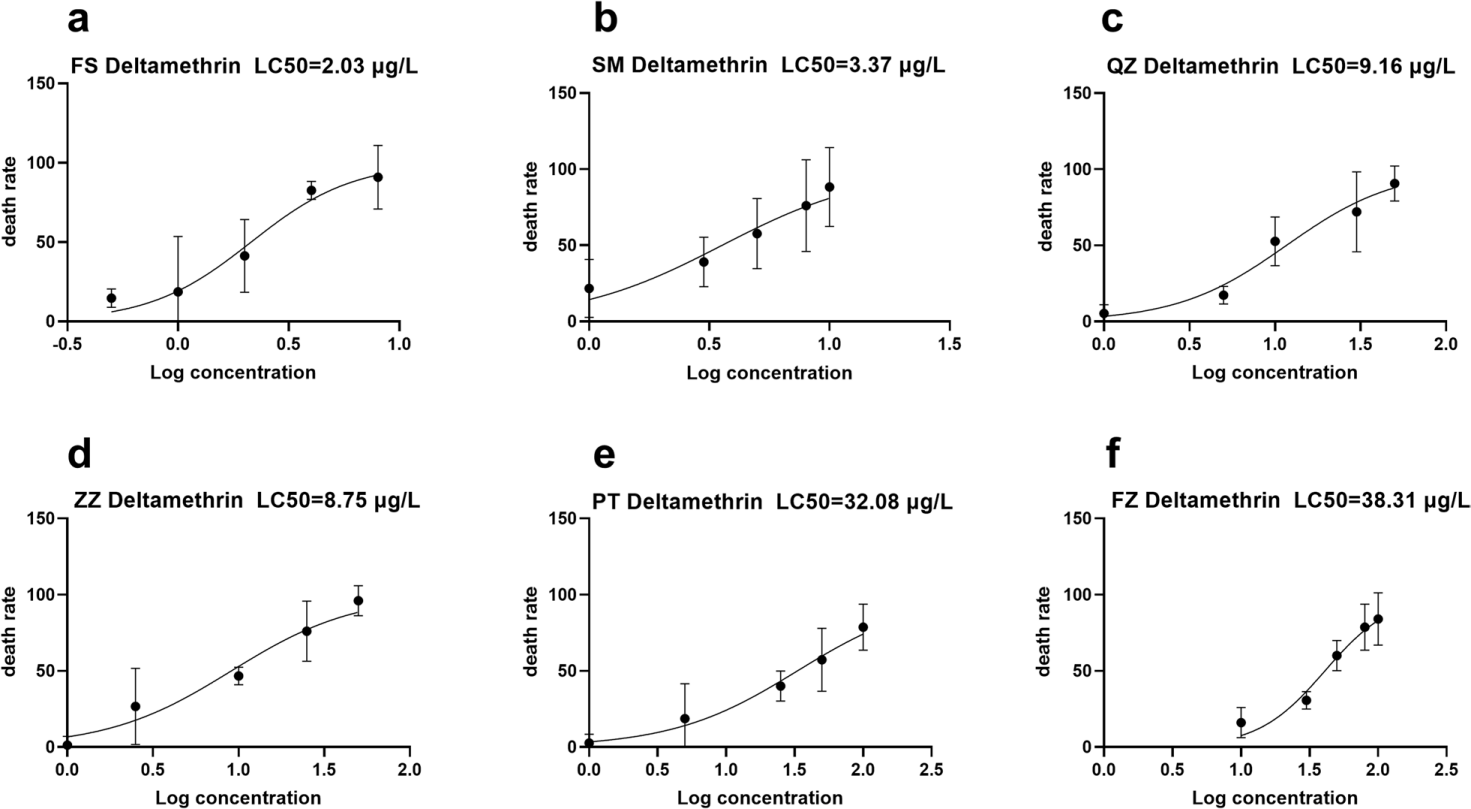
Resistance of *Aedes albopictus* to deltamethrin. **a** FS population, **b** SM population, **c** QZ population, **d** ZZ population, **e** PT population, **f** FZ population. FS, Foshan; LC_50_, half-maximal lethal concentration; SM, Sanming; QZ, Quanzhou; ZZ, Zhangzhou; PT, Putian, FZ, Fuzhou

The sensitive population of *Ae. albopictus* (FS population) exhibited an LC_50_ of 2.03 μg/l against deltamethrin. In comparison the SM, QZ, ZZ, PT and FZ populations of Fujian Province had an LC_50_ of 3.37, 9.16, 8.75, 33.08 and 38.31 μg/l, respectively. As shown in Fig. 1, *Ae. albopictus* of the SM population was relatively sensitive to deltamethrin. The ZZ and QZ populations exhibited RR of approximately 4.31 and 4.51, respectively, indicating sensitivity but nearing a moderate resistance level. The PT and FZ *Ae. albopictus* populations were found to have developed high resistance to deltamethrin, with resistance coefficients of approximately 15.80 and 18.87, respectively. The resistance levels are shown in Table 1, and the raw data are provided in Additional file 2: Data file S2.

**Table 1:** Levels of resistance of *Aedes albopictus* to deltamethrin and pyriproxyfen in various cities of Fujian Province

Region (abbreviation)	Deltamethrin		PPF	
	LC_50_ (95% CI) (μg/L)	RR	IE_50_ (95% CI) (μg/L)	RR
Foshan (FS)	2.03 (1.26, 3.29)	1.00	0.13 (0.07, 0.23)	1.00
Sanming (SM)	3.37 (1.77, 5.17)	1.66	0.14 (0.07, 0.24)	1.08
Quanzhou (QZ)	9.16 (7.78, 10.71)	4.51	0.15 (0.04, 0.35)	1.15
Zhangzhou (ZZ)	8.75 (3.23, 20.88)	4.31	0.37 (0.32, 0.43)	2.85
Putian (PT)	32.08 (25.56, 40.61)	15.80	0.37 (0.11, 0.80)	2.85
Fuzhou (FZ)	38.31 (24.23, 54.33)	18.87	0.47 (0.41, 0.55)	3.62

*CI *Confidence interval,* IE*_*50*_, half-maximal inhibition of emergence,* LC*_*50*_ half-maximal lethal concentration,* PPF* pyriproxyfen,* RR *resistance ratio

### Resistance of *Ae. albopictus* to PPF in various cities in Fujian Province

**Fig. 2:**
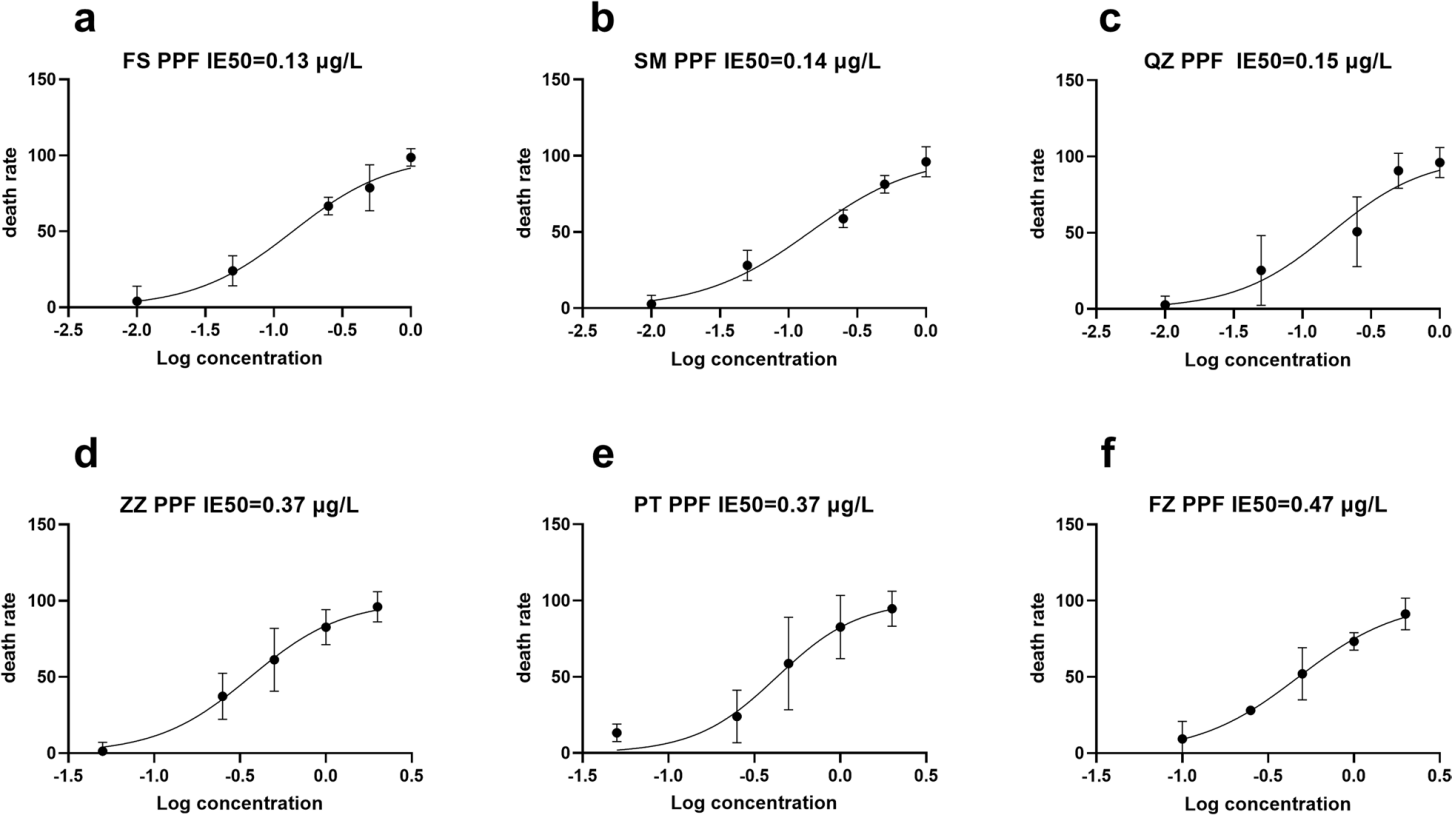
Resistance of *Ae. albopictus* to PPF. **a** FS population. **b** SM population. **c** QZ population. **d** ZZ population. **e** PT population. **f** FZ population. FS, Foshan; IE_50_, half-maximal inhibition of emergence; SM, Sanming; QZ, Quanzhou; ZZ, Zhangzhou; PT, Putian, FZ, Fuzhou

The IE_50_ of the FS-sensitive population of *Ae. albopictus* to PPF was 0.13 μg/l. The IE_50_ of the SM, QZ and ZZ populations was 0.14, 0.15 and 0.37 μg/l, respectively, whereas the PT and FZ populations had IE_50_ values of 0.37 and 0.47 μg/l, respectively. As shown in Fig. 2, the SM and QZ populations of *Ae. albopictus* were more sensitive to PPF. In other regions, *Ae. albopictus* has developed varying levels of resistance to PPF, with resistance levels ranging from 2.85-fold that of the ZZ population, 2.85-fold that of the PT population to 3.62-fold that of the FZ population. The resistance levels are shown in Table 1, and the raw data are included in Additional File 2: Data file S2 (bioassay experiments).

### Mutation results on three representative mutation sites (V1016V, I1532I, F1534F) on the VGSC gene

**Fig. 3:**
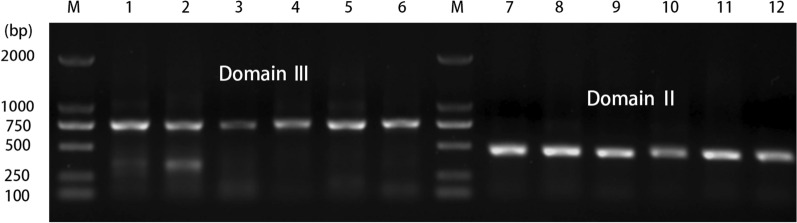
PCR amplification electrophoresis map of Domain II and Domain III fragments of the *Ae. albopictus* VGSC gene. Lanes: M, DNA marker; 1–6, amplification products of the Domain III fragment in FS, QZ, PT, ZZ, FZ and SM, respectively; 7–12, amplification products of the Domain II fragment in FS, QZ, PT, ZZ, FZ and SM, respectively. FS, Foshan; SM, Sanming; QZ, Quanzhou; ZZ, Zhangzhou; PT, Putian, FZ, Fuzhou; VGSC, voltage-gated sodium channe

To perform comparative analysis, we amplified and sequenced Domains II and III of the VGSC gene. The PCR products of the VGSC gene Domain II were about 500 bp, and those of Domain III were about 750 bp. The results from agarose gel electrophoresis are shown in Fig. 3. Stable signal fragments were extracted for comparative analysis in which we aligned the obtained sequences with the coding regions II and III of the VGSC gene in *Ae. albopictus* using the BLAST algorithm on NCBI. The partial sequence consistency was found to be 99.00% (matching with accession numbers KC152045.1 and KC152046.1), confirming that our sequence represents a partial fragment of the VGSC gene in *Ae. albopictus*.

Notably, the results showed that there were certain degrees of mutations at V1016, I1532 and F1534, with base changes leading to corresponding amino acid substitutions. These mutations included: (i) V1016, in which the GTA codon mutates to TGT, resulting in the replacement of valine (V) with cysteine (C), and (ii) I1532, in which the ATC codon encoding isoleucine (I) showed mutations into TTC, TAC, and ACC; these changes correspond to phenylalanine (F), tyrosine (Y), and threonine (T), respectively. (iii) A third mutation occurred at F1534; this codon showed the most diverse mutations, with the TTC codon encoding phenylalanine (F) mutating into TCC/TCG, TGC, TTG and TTG; these changed codons encode serine (S), C, leucine (L) and F (synonymous mutations), respectively. Detailed genotype sequencing maps are should in Additional file 3: Figure S1.

As shown in Table 2, the sample sizes of V1016 in each region are all 30. For I1532 and F1534 in the SM, QZ, ZZ, PT, FZ and FS populations, the sample sizes are 30, 26, 32, 30, 36 and 33, respectively. We observed the a number of non-synonymous mutations in the VGSC gene of *Ae. albopictus*.

V1016C, a mutation which results in the replacement of V with C, was found in the FZ population, with a mutation frequency of 10.00%.

 A variety of possible non-synonymous mutations were found in all populations, with the exception of the SM population at I1532. I1532Y and I1532F are two possible non-synonymous mutations that occurred in the FZ population at I1532, with mutation frequencies of 8.33% and 16.67%, respectively.

 In all populations, diverse non-synonymous mutations were observed at F1534. Specifically, the PT population and the FZ population exhibited the following mutations: F1534S, with mutation frequencies of 90.00% and 33.33%, respectively, and F1534L, with mutation frequencies of 10.00% and 7.69%, respectively.

**Table 2:** Genotype and frequency of three mutation sites (V1016, I1532, and F1534) in the voltage-gated sodium channel gene of *Aedes albopictus* collected in a number of cities of Fujian Province

Region (abbreviation)	V1016		I1532				F1534			
	V1016V	V1016C	I1532I	I1532Y	I1532T	I1532F	F1534F	F1534S	F1534L	F1534C
Sanming (SM)	30 (100.00%)	/^a^	30 (100.00%)	/	/	/	18 (60.00%)	9 (30.00%)	3 (10.00%)	/
Quanzhou (QZ)	30 (100.00%)	/	10 (38.46%)	10 (38.46%)	2 (7.69%)	4 (15.38%)	/	22 (84.62%)	2 (7.69%)	2 (7.69%)
Zhangzhou (ZZ)	30 (100.00%)	/	28 (87.50%)	4 (12.50%)	/	/	24 (75.00%)	8 (25.00%)	/	/
Putian (PT)	30 (100.00%)	/	27 (90.00%)	/	/	3 (10.00%)	/	27 (90.00%)	3 (10.00%)	/
Fuzhou (FZ)	27 (90.00%)	3 (10.00%)	27 (75.00%)	3 (8.33%)	/	6 (16.67%)	21 (58.33%)	12 (33.33%)	3 (8.33%)	/
Foshan (FS)	30 (100.00%)	/	24 (72.73%)			9 (27.27%)	33 (100.00%)	/	/	/

The data in the table show the frequencies of each genotype corresponding to different genotypes at each mutation site. Numbers in parentheses indicate the proportion of each genotype

V1016V, V1016C, I1532I, I1532Y, I1532T, I1532F, F1534F, F1534S, F1534L and F1534C represent genotypes, where V, C, I, Y, T, F, S and L denote valine, cysteine, isoleucine, tyrosine, threonine, phenylalanine, serine and leucine, respectively

^a^“/” indicates the absence of mutation at that site

### Molecular docking results of PPF and its metabolites 4'-OH-PPF with CYPs

**Fig. 4:**
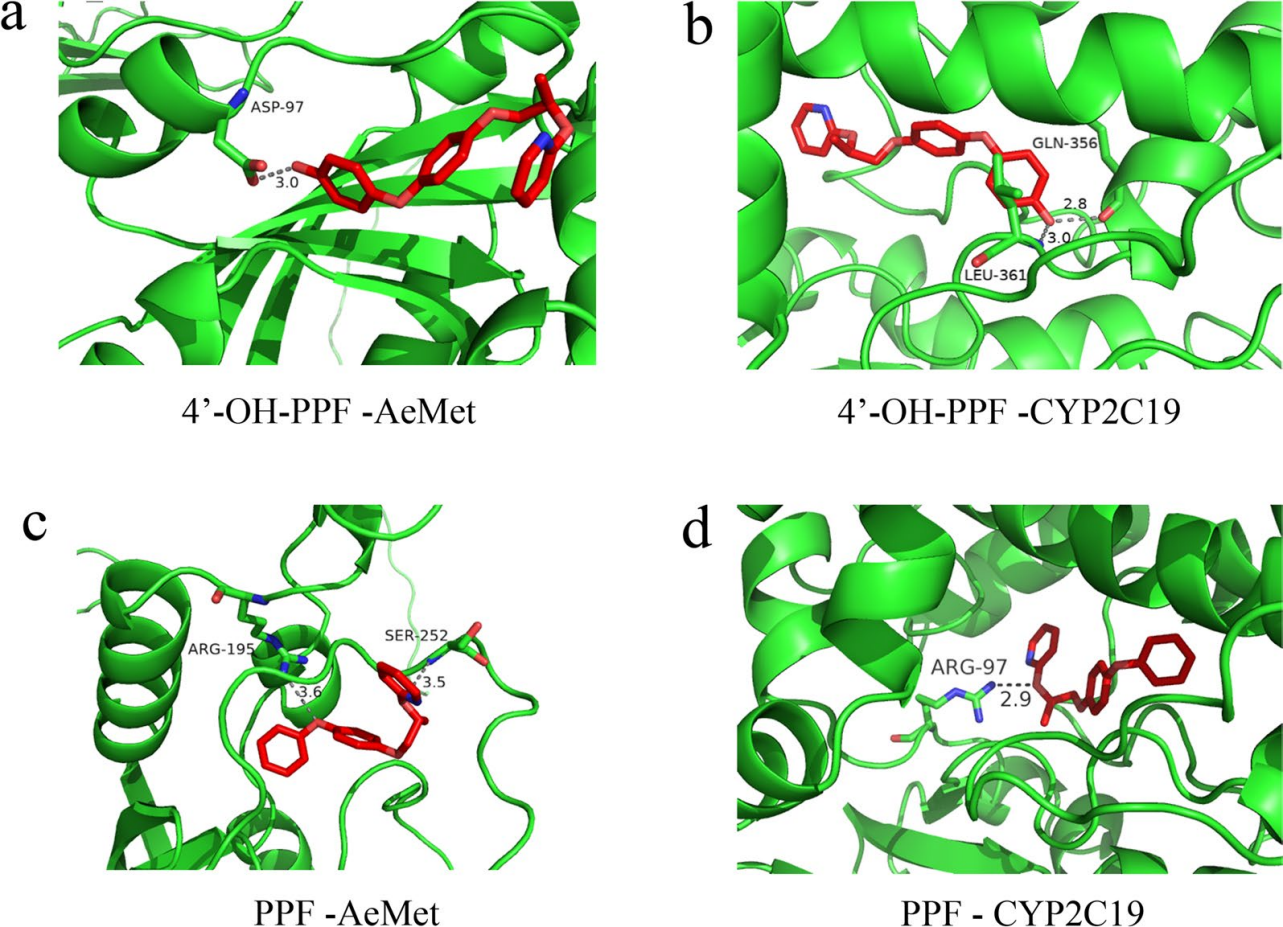
Molecular docking results. **a** Molecular docking of 4’-OH-PPF with the AeMet receptor. **b** 4′-OH-PPF with the CYP2C19 receptor. **c** PPF with the AeMet receptor. **d** PPF with the CYP2C19 receptor. The schematic represents the backbone structure of the cytochrome P450 or AeMet. The residues involved in the interaction are shown with a stick model. The oxygen and nitrogen atoms shown in red and blue, respectively. The interactions are labeled by dotted lines. AeMet receptor, *Ae. albopictus* methoprene-tolerant receptor; CYP2C19, cytochrome P450 2C19; PPF, pyriproxyfen; 4′-OH-PPF, metabolite of pyriproxyfen

Molecular docking results revealed that PPF and its metabolite 4'-OH-PPF bound to the *Ae. albopictus* methoprene-tolerant receptor (AeMet receptor) and CYP2C19. 4'-OH-PPF was located in the groove of the N-terminus of AeMet, and the side chain of Asp97 formed a hydrogen bond with length 3.0 Å with the methyl group at the end of 4'-OH-PPF (Fig. 4a). The terminal methyl group of 4'-OH-PPF was coordinated by a network of hydrogen bonds to the main chain amide group of Leu361 and the carboxylate oxygen atom from Gln356 (Fig. 4b). Different from 4'-OH-PPF, the skeleton of PPF was stabilized by Arg195 and Ser252 from AeMet via hydrogen bonds (Fig. 4c), while PPF interacted with the 2C19 region of P450 at Arg97 with a bond length of 2.9 Å (Fig. 4d). We also performed molecular docking of PPF and its metabolite 4'-OH-PPF with CYP2C9 and CYP3A4, and the results showed that they all had varying degrees of binding (Additional file 4: Figure S2). The results of the molecular docking and larvae bioassays provided insights into the capacity of these enzymes to metabolize diverse insecticides, which could impact vector control strategies.

## Discussion

Fujian Province is a high-incidence area of dengue fever in China, with *Ae. albopictus* serving as the primary vector for dengue fever transmission. In recent years, the number of dengue fever cases has escalated due to frequent importation from foreign countries and neighboring provinces. This has resulted in an increase in the incidence rate, frequent local outbreaks and expanded distribution [29, 30].

Our study revealed varying degrees of resistance to deltamethrin and PPF in *Ae. albopictus* populations across different regions of Fujian Province. Notably, *Ae. albopictus* populations in the PT and FZ regions exhibited relatively higher resistance levels, most likely associated with the local dengue fever epidemic and the use of insecticides in these two areas. We also detected three pyrethroid resistance-related alleles in the *Ae. albopictus* VGSC gene: V1016V, I1532I and F1534F, as well as several non-synonymous mutations. These mutations might affect the binding ability of deltamethrin to sodium channels, thus reducing the insecticidal effect of deltamethrin.

The effect of the V1016 mutation has long been identified in *Aedes aegypti* [31–33]. In our study, the V1016C mutation was exclusively found in the FZ population of *Ae. albopictus*, which is consistent with previous results, indicating that this mutation is rare in *Ae. albopictus* in China [11]. However, Zhou et al. [34] reported contrasting results for *Ae. albopictus* specimens collected from various districts of Beijing from 2016 to 2017, revealing a significantly higher mutation rate at V1016 compared to F1534. This discrepancy may be related to the historical use of insecticides in Fujian Province. Also in our study, we observed mutations at I1532 across all populations, including the susceptible population (FS). Additionally, two potential mutations (I1532F and I1532Y) were detected in the FZ population. While the I1532F mutations in susceptible populations may not be associated with deltamethrin resistance, the role of the I1532Y mutation in the FZ population increased deltamethrin resistance in *Ae. albopictus* and deserves further exploration. The QZ population exhibited the I1532Y mutation. The I1532T mutation has been previously reported in *Ae. albopictus* from Italy [20] and different areas of China [19, 30, 35].

Mutations at the F1534 target site were detected in all populations of *Ae. albopictus*, with varying proportions of F1534S, F1534L and F1534C mutations. These mutations likely contribute to the resistance of *Ae. albopictus* to deltamethrin. This finding is in agreement with results reported by Kasai et al., who first identified F1534C mutations in the *Ae. albopictus* population in Singapore [21]. Additionally, Xu et al. identified F1534S, F1534C and F1534L mutations in wild populations across different countries [20]. Recently, Guo et al. utilized CRISPR/Cas9 technology to knock out the F1534S mutant region of the VGSC gene in *Ae. albopictus*, confirming the pivotal role of this mutation in deltamethrin resistance [36]. A mutation detection study of the *kdr* gene in *Ae. albopictus* collected from dengue surveillance sites in Fujian Province in 2015 and 2017 showed mutations at both I1532 and F1534 [37]. Our study also found mutations at I1532 in the QZ, ZZ, PT and FZ populations. Mutations at F1534 were found in the SM, QZ, ZZ, PT and FZ populations. Additionally, a non-synonymous mutation was detected at the V1016C allele of the FZ population. Further studies are needed necessary to determine the precise relationship between these mutations and insecticide resistance.

For cross-resistance to deltamethrin and PPF, certain P450 enzymes (such as CYP6M2, -6P2, -6P3, -6P4, -6P5 and -9J5) have been implicated in pyrethroid resistance and cross-resistance to PPF in *Anopheles gambiae* [38]. In *Culex pipiens pallens*, the relative expression level of the *Culex pipiens pallens methoprene-tolerant* (*CpMet*) gene in the resistance population significantly decreased to 43.59% of that in the susceptible population. Further investigation revealed a close link between the reduced expression of the *Met* gene and increased resistance level [39]. Although cross-resistance between pyrethroids and PPF exists in the *Ae. albopictus* population [11], the specific underlying mechanism remains unknown. Our study provides molecular-level evidence that the PPF metabolite 4′-OH-PPF binds to the AeMet receptor. However, the predicted protein structure of the AeMet receptor might have contributed to the low number of hydrogen bonds observed in the results. Similar challenges were encountered with the 4′-OH-PPF and P450s. Moving forward, we hope that future research, potentially by structural biologists, will elucidate the structure of mosquito CYP enzymes. Additionally, we look forward to experimental validation of the metabolic functions of CYP2C19, CYP2C9 and CYP3A4.

The results of the PPF bioassay demonstrated that the resistance coefficient of PPF in the FZ and PT populations exceeded that of the susceptible population. Furthermore, upon analyzing the association with deltamethrin resistance, we found a positive correlation between deltamethrin resistance and PPF resistance in the wild *Ae. albopictus* populations in Fujian. However, an interesting phenomenon was also observed in the QZ area. Initially, due to the small population size during the early testing stage, the QZ population exhibited low resistance to deltamethrin and PPF. After the population had reproduced to the experimental dosage and larvae across multiple generations were used, we noted that the resistance levels increased. In *Ae. aegypti*, the QZ population exhibited the F1534C mutation and displayed pyrethroid resistance [40–42]. The F1534C mutation was found to reduce the channel sensitivity to pyrethroids in *Xenopus* oocytes, further confirming the role of the F1534C gene mutation in insecticide resistance [43]. To validate these findings, future research should involve collecting a new population of *Ae. albopictus* from the QZ area; this will enable verification of the impact of the F1534C mutation on deltamethrin and PPF resistance.

## Conclusions

In this study, multiple populations of *Ae. albopictus* from Fujian Province exhibited varying degrees of resistance to deltamethrin and PPF, with higher resistance levels observed in populations collected from the PT and FZ areas. Among the non-synonymous mutations detected in the VGSC gene, those mutations at F1534 likely play a significant role in *Ae. albopictus* resistance to deltamethrin. We also found that *Ae. albopictus* exhibited a trend of cross-resistance to deltamethrin and PPF, which is possibly linked to the impact of both insecticides on the insect nervous system or their reliance on P450 enzyme metabolism for detoxification. To address this challenge, future efforts should focus on: (i) strengthening environmental management and mosquito control; (ii) rational use of insecticide types and appropriate dosages; and (iii) vigilance against the development of higher levels of cross-resistance, particularly in the PT and FZ regions. By implementing these strategies, it will be possible to enhance vector control and mitigate the impact of insecticide resistance in *Ae. albopictus* populations.

## Supplementary Information

**Additional file 1: Data file S1.** Excel spreadsheet showing sampling information on the collection of *Ae. albopictus *in Fujian Province.

**Additional file 2: Data file S2.** Excel spreadsheet showing the bioassay experiments.

**Additional file 3: Figure S1.** Genotype sequencing map of three sites (V1016V, I1532I, and F1534F) in the VGSC gene of *Ae. albopictus* in various cities of Fujian Province.

**Additional file 4: Figure S2**. Molecular docking results for two additional CYP450 receptors: CYP2C9 and CYP3A4.** a** Molecular docking of 4’-OH-PPF with CYP2C9 receptor.** b** PPF with CYP2C9 receptor.** c** 4’-OH-PPF with CYP3A4 receptor.** d** PPF with CYP3A4 receptor. The schematic represents the backbone structure of P450. The residues involved in the interaction are shown with a stick model. The oxygen and nitrogen atoms shown in red and blue, respectively. The interactions are labeled by dotted lines.

## Data Availability

The datasets used and/or analyzed during the current study are available from the corresponding author on reasonable request.

## Abbreviations

AeMet: *Aedes albopictus* methoprene-tolerant receptor
C: Cysteine
*CpMet*: *Culex pipiens pallens methoprene-tolerant*
F: Phenylalanine
FS: Foshan
FZ: Fuzhou
H: Histidine
I: Isoleucine
IE_50_: Half-maximal inhibition of emergence
JH: Juvenile hormone
KA: Association constant
*kdr*: Knockdown resistance
L: Leucine
LC_50_: Half-maximal lethal concentration
PPF: Pyriproxyfen
PT: Putian
QZ: Quanzhou
RR: Resistance ratio
S: Serine
SM: Sanming
T: Threonine
V: Valine
VGSC: Voltage-gated sodium channel
Y: Tyrosine
ZZ: Zhangzhou
