# Supplementary figures and images for: Study on the cross-resistance of Aedes albopictus (Skuse) (Diptera: Culicidae) to deltamethrin and pyriproxyfen
Source: Parasit Vectors. 2024 Sep 27;17:403. doi: 10.1186/s13071-024-06485-1 (PMC11438187; doi:10.1186/s13071-024-06485-1)

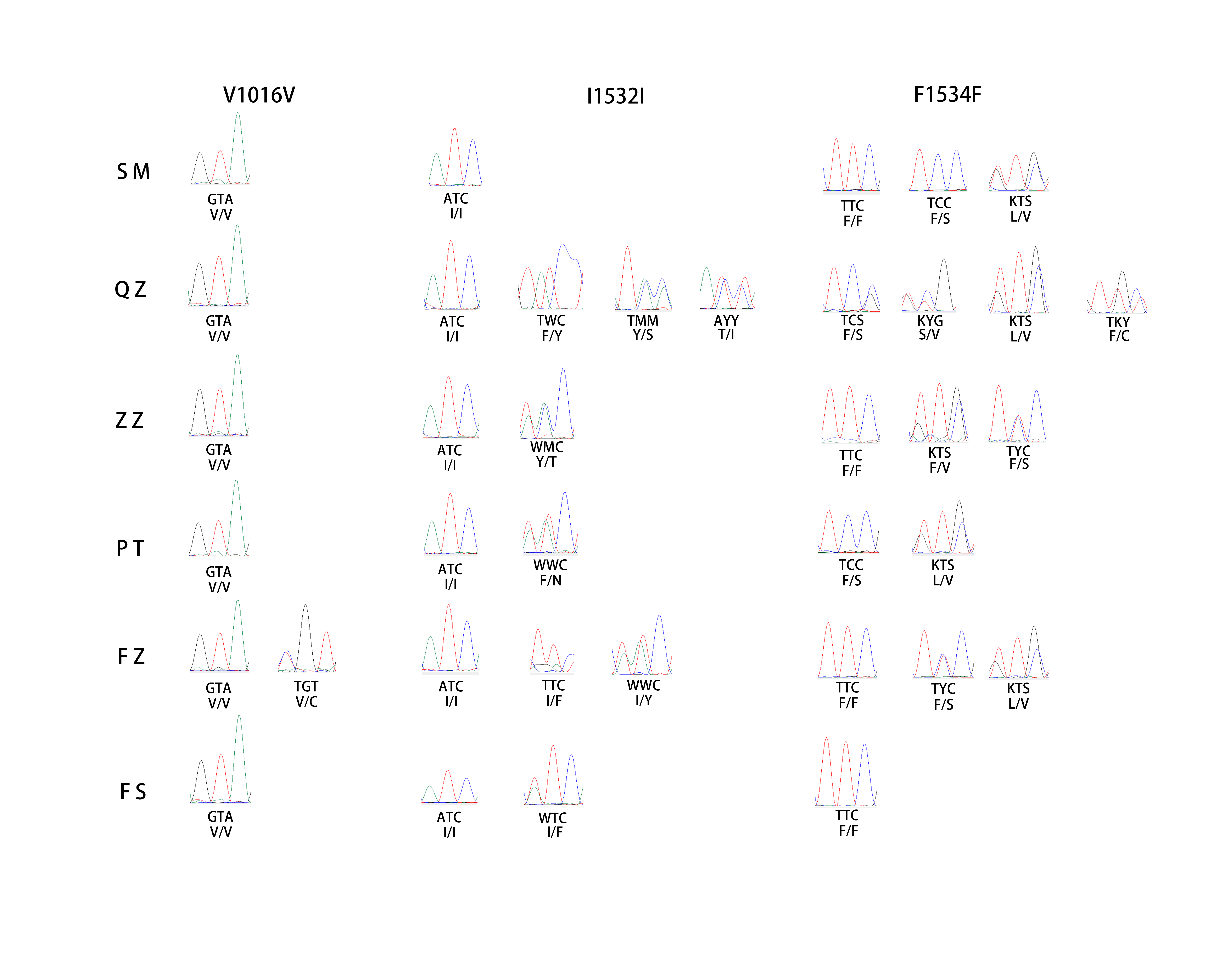

Supplement: Supplementary file 3 — Additional file 3: Figure S1. Genotype sequencing map of three sites (V1016V, I1532I, and F1534F) in the VGSC gene of Ae. albopictus in various cities of Fujian Province. [file 13071_2024_6485_MOESM3_ESM.tif]

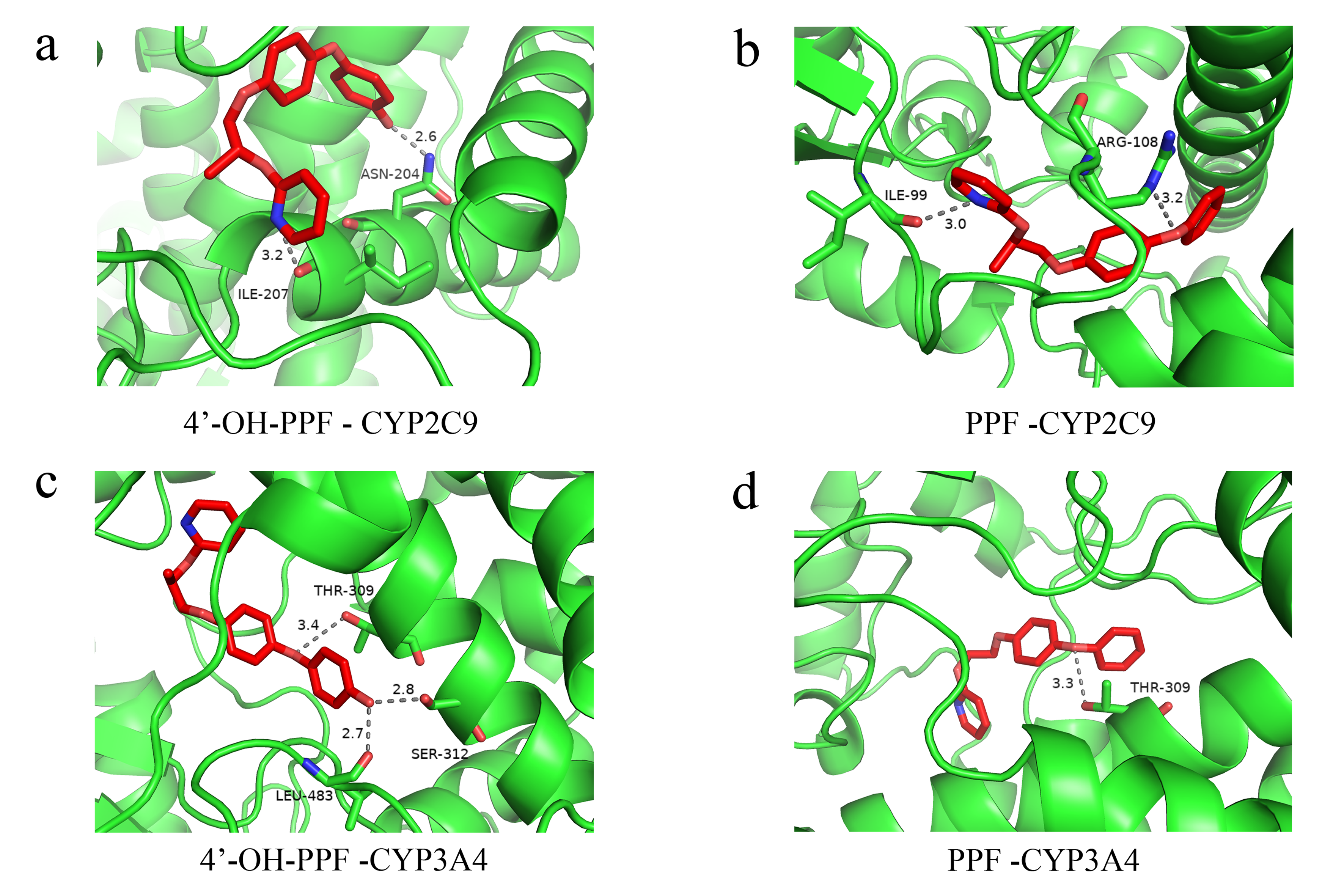

Supplement: Supplementary file 4 — Additional file 4: Figure S2. Molecular docking results for two additional CYP450 receptors: CYP2C9 and CYP3A4. a Molecular docking of 4’-OH-PPF with CYP2C9 receptor. b PPF with CYP2C9 receptor. c 4’-OH-PPF with CYP3A4 receptor. d PPF with CYP3A4 receptor. The schematic represents the backbone structure of P450. The residues involved in the interaction are shown with a stick model. The oxygen and nitrogen atoms shown in red and blue, respectively. The interactions are labeled by dotted lines. [file 13071_2024_6485_MOESM4_ESM.tif]
